# Whole Exome Sequencing Identifies a Heterozygous Variant in the Cav1.3 Gene *CACNA1D* Associated with Familial Sinus Node Dysfunction and Focal Idiopathic Epilepsy

**DOI:** 10.3390/ijms232214215

**Published:** 2022-11-17

**Authors:** Susanne Rinné, Birgit Stallmeyer, Alexandra Pinggera, Michael F. Netter, Lina A. Matschke, Sven Dittmann, Uwe Kirchhefer, Ulrich Neudorf, Joachim Opp, Jörg Striessnig, Niels Decher, Eric Schulze-Bahr

**Affiliations:** 1 Institute of Physiology and Pathophysiology, Vegetative Physiology, University of Marburg, 35037 Marburg, Germany; 2 Institute for Genetics of Heart Diseases (IfGH), University Hospital Muenster, 48149 Muenster, Germany; 3 Department of Pharmacology and Toxicology, Center for Molecular Biosciences, University of Innsbruck, 6020 Innsbruck, Austria; 4 Institute of Pharmacology and Toxicology, University Hospital Muenster, 48149 Muenster, Germany; 5 Zentrum für Kinder-und Jugendmedizin, Klinik für Kinderheilkunde III-Bereich Kardiologie, University Hospital Essen, 45147 Essen, Germany; 6 Ev. Krankenhaus Oberhausen, 46047 Oberhausen, Germany

**Keywords:** calcium channel, Cav1.3, sinoatrial node dysfunction, focal idiopathic epilepsy

## Abstract

Cav1.3 voltage-gated L-type calcium channels (LTCCs) are involved in cardiac pacemaking, hearing and hormone secretion, but are also expressed postsynaptically in neurons. So far, homozygous loss of function mutations in *CACNA1D* encoding the Cav1.3 α_1_-subunit are described in congenital sinus node dysfunction and deafness. In addition, germline mutations in *CACNA1D* have been linked to neurodevelopmental syndromes including epileptic seizures, autism, intellectual disability and primary hyperaldosteronism. Here, a three-generation family with a syndromal phenotype of sinus node dysfunction, idiopathic epilepsy and attention deficit hyperactivity disorder (ADHD) is investigated. Whole genome sequencing and functional heterologous expression studies were used to identify the disease-causing mechanisms in this novel syndromal disorder. We identified a heterozygous non-synonymous variant (p.Arg930His) in the *CACNA1D* gene that cosegregated with the combined clinical phenotype in an autosomal dominant manner. Functional heterologous expression studies showed that the *CACNA1D* variant induces isoform-specific alterations of Cav1.3 channel gating: a gain of ion channel function was observed in the brain-specific short *CACNA1D* isoform (Cav1.3_S_), whereas a loss of ion channel function was seen in the long (Cav1.3_L_) isoform. The combined gain-of-function (GOF) and loss-of-function (LOF) induced by the R930H variant are likely to be associated with the rare combined clinical and syndromal phenotypes in the family. The GOF in the Cav1.3_S_ variant with high neuronal expression is likely to result in epilepsy, whereas the LOF in the long Cav1.3_L_ variant results in sinus node dysfunction.

## 1. Introduction

Inherited or idiopathic sinoatrial node dysfunction (SND) is an uncommon disorder that includes sinus bradycardia, sinoatrial and atrio-ventricular nodal conduction defects and sometimes supraventricular arrhythmias including atrial fibrillation. Mutations in several cardiac ion channel or associated regulatory genes, including *HCN4* [1], *SCN5A* [2], *KCNJ5* [3], *KCNJ3* [4], *TRPM4* [5,6], *GNB2* [7] and *GNB5* [8,9] have been associated with autosomal dominant SND and/or related cardiac conduction disorders. However, in many SND patients, the underlying cause might be non-genetic, but be related to extrinsic causes and phenocopies (e.g., high vagal tone, negative chronotropic medication, hypothyroidism, hypothermia or cardiac denervation). Overall, a familial occurrence of isolated sinus bradycardia in particular is uncommon, but has long been recognized [10], and sometimes SND is also seen in the setting of cardiomyopathy [11], congenital heart disease or a syndromal disorder with extracardiac features [8,12].

Apart from the classical cardiac pacemaker channel gene *HCN4*, which contributes to the “funny current” *I*_f_ (a mixed conductance with an inward current carried by Na^+^, activated at hyperpolarized membrane potentials), acetylcholine-activated potassium currents (*I*_K,ACh_) [13] and L- and T-type Ca^2+^ channels (LTCC and TTCC) [14,15] are essential for the cardiac pacemaking in sino-nodal cells. The L-type Ca^2+^ currents (*I*_Ca,L_) in the sinoatrial node depend on voltage-gated Cav1.3 (gene: *CACNA1D*) and Cav1.2 (gene: *CACNA1C*) channels, whereas in the working myocardium, *I*_Ca,L_ is mediated by Cav1.2, only [16]. Cav1.3 channels activate at more negative membrane potentials and are thus suitable to contribute to diastolic depolarization in SAN cells [17].

LTCCs and TTCCs are macromolecular complexes consisting of a central ion conducting protein (the α_1_ subunit) and (in the case of LTCCs) additional accessory channel subunits (α_2_δ, β and γ), which modulate their trafficking and gating properties. The electrophysiological properties of Cav1.3 are mainly determined by the α_1_ subunit, a transmembrane protein consisting of four homologous repeats (DI-DIV) of six transmembrane segments that include the four voltage sensors and pore loops to form the functional channel. Transcripts of *CACNA1D* are expressed in many tissues, including the sinoatrial and atrio-ventricular (AV) node [16], the brain [18] and cochlea [19] and expression of functionally distinct Cav1.3 α_1_-subunit isoforms differs between tissues [20]. In 2000, LTCC deficient mice were reported with deafness due to the complete absence of L-type calcium currents in cochlear inner hair cells and degeneration of outer and inner hair cells [21]. In addition, these mice showed bradycardia, indicating a relevant physiologic role of Cav1.3 channels for both regular hearing and sinoatrial node function. In 2010 the first homozygous loss-of-function mutation (p.Gly403dup) in the *CACNA1D* gene was described in a consanguineous Pakistani family with congenital deafness and sick sinus syndrome; this complex and syndromal disease was termed ‘SANDD’ (sinoatrial node dysfunction and deafness) [12]. Very recently, an additional homozygous mutation (p.Ala376Val) in the *CACNA1D* was identified in a SANDD family [22]. In addition, a few *de novo* or somatic *CACNA1D* gene variants have been described in primary hyperaldosteronism, in aldosterone-producing adenomas and/or neurologic diseases, including epileptic seizures, autism spectrum disorder, developmental delay and attention deficit hyperactivity disorder (ADHD) -like symptoms [23,24,25,26]. Typically, these variants cause changes in channel gating, enabling channel gain-of-function. This indicates a prominent but diverse function of the Cav1.3 L-type calcium channel in regulation of cellular excitability in various tissues.

Here, we describe a heterozygous non-synonymous variant (p.Arg930His) in the *CACNA1D* gene identified in a three-generation Turkish family with a phenotype composed of cardiac and neurologic symptoms including sinus bradycardia, atrio-ventricular block (AVB), idiopathic epilepsy and learning disability, but without signs for congenital deafness. The trait of inheritance was autosomal dominant. Heterologous functional studies in various cellular models showed that a mutant *CACNA1D* induces an isoform-specific alteration of Cav1.3 channel gating characterized by a gain of channel function in the brain-specific short isoform and a loss of channel function observed for the cardiac-specific long isoform.

## 2. Results

### 2.1. Clinical Characteristics of CACNA1D R930H Variant Carriers

**Figure 1:**
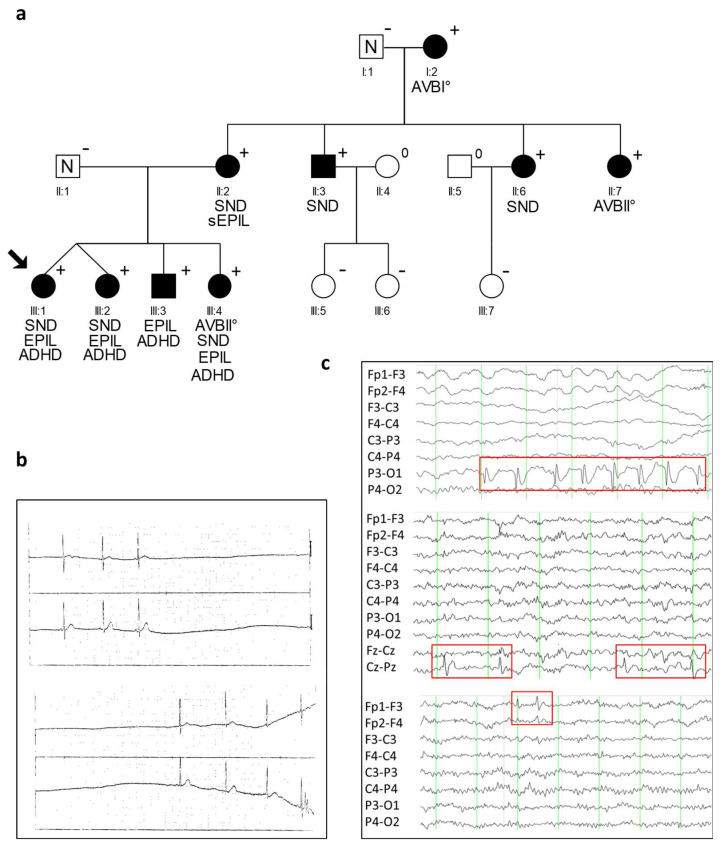
Identification of the heterozygous CACNA1D variant R390H. (**a**) Pedigree of the family with a combined clinical phenotype of cardiac and extra-cardiac symptoms with a heterozygous *CACNA1D* variant (p.Arg930His; shortly: R930H) and autosomal dominant syndromic form of SND and epilepsy. Men are denoted by squares, woman by circles. Filled symbols represent clinically affected family members; N indicates family members without a diagnosis of SND or epilepsy, + presence of the heterozygous variant R930H, − absence of the heterozygous variant R930H, 0 not tested. The proband is indicated by an arrow. Characteristics of cardiac and neurologic phenotypes are indicated below (SND, sinus node dysfunction; AVB, atrio-ventricular block; Epil., epilepsy; sEpil., suspected epilepsy; ADHD, attention deficit hyperactivity disorder). (**b**) ECG of proband (III:1) at age 5 years with documented sinus arrest, and (**c**) EEG of 3 *CACNA1D* variant carriers demonstrating occurrence of sharp waves with different localization. Above: EEG of family member III:I at the age of five shows occipital sharp waves (P3-O1) and bulbus artifacts (Fp1 and Fp2). Middle: EEG of family member III:2 at the age of 10 with central sharp waves (Cz-Pz). Down: EEG of index case (III:1) at the age of 10 years with left frontal sharp waves (Fp1-F3). The red boxes indicate abnormal EEG changes. Fp = most frontal leads; F = frontal; C = central; *p* = parietal; O = occipital.

The index proband of a Turkish family, a 5-year-old girl, presented with syncope accompanied by epileptic seizures (Figure 1a). Cardiac examination detected pronounced SND with severe bradycardia (Figure 1b). She received a pacemaker, however the episodes of epileptic seizures persisted, and a diagnosis of epilepsy was confirmed by the occurrence of sharp waves in several EEG analyses (Table 1, Figure 1c). A similar neurologic phenotype accompanied by sinus arrhythmia was also observed for the monozygotic twin sister, compatible with a genetic cause for the disease.

Re-evaluation of ECG data from all family members available revealed six additional family members with varying clinical cardiac manifestation from SND to AVB (Figure 1a, Table 1). In contrast, episodes of epileptic seizures were documented in four children; three of them were also affected by cardiac conduction disease. The epilepsy in all four children showed the following characteristics: (1) Focal seizures with impairment or loss of consciousness, (2) normal MRI scans, (3) a benign course with freeness of seizures and EEG abnormalities between the age of 5 to 13 years, and (4) focal epileptiform discharges with attenuation in drowsiness and sleep. Interestingly, the location of these abnormal EEG changes, mainly sharp waves, was not uniform, but varied between affected family members and also between EEGs of a specific variant carrier at different ages. This demonstrates a general possibility of all cortical cells to generate epileptic potentials, which is typical for epileptic syndromes of genetic origin. The patient (III:3) had lateralized occipital sharp waves, which were accompanied by synchronous blinking of the eyes, and which was sometimes blocked by eye opening (fixation-off phenomenon). Due to these features, we classified the epilepsy as “late onset childhood occipital epilepsy (Gastaut-type)”. All four siblings had an additional diagnosis of attention deficit hyperactivity disorder (ADHD) and were treated with stimulants.

The mother of the four siblings (II:2) experienced one seizure with loss of consciousness while driving her car. Her EEG was normal. In the other family members, no indications for a neurologic disorder were described in clinical records. In two of them (II:3, II:7), an EEG could be obtained and showed no abnormalities. None of the family members were deaf or had any signs for an impairment of the auditory system.

### 2.2. Identification of a Heterozygous Non-Synonymous Variant (p.R930H) in the CACNA1D Gene Cosegregating in Syndromic Sinus Bradycardia and Epilepsy

**Figure 2:**
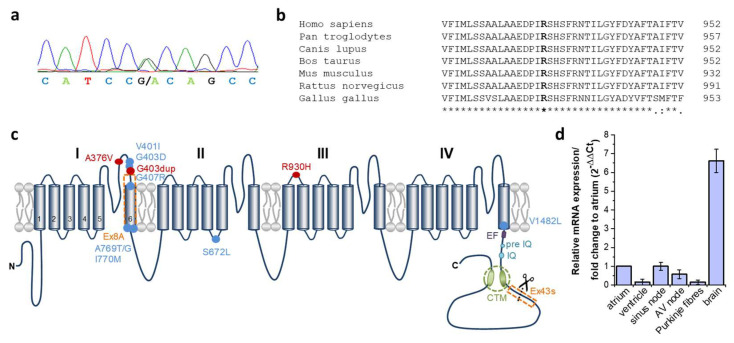
Conservation and localization of the heterozygous CACNA1D variant R390H. (**a**) Electropherogram of the proband (III:1) with the heterozygous *CACNA1D* nucleotide variant c.2789G>A (NM_000720.3). The peaks represent each base in the gDNA sequence (red, thymine; green, adenine; black, guanine and blue, cytosine). (**b**) Multiple sequence alignment of the human CACNA1D protein region encompassing arginine 930 with orthologous protein sequences. Identical amino acids are indicated by an asterisk and highly conserved amino acids by a colon in the lower lane. The mutated amino acid residue is indicated in bold. (**c**) The predicted topology of the Cav1.3 channel and structure of the human transcript variants used in functional studies of Cav1.3. Cav1.3 α_1_-subunit comprised of four structurally homologous domains (I, II, III, IV), each containing six transmembrane spanning domains (S1–S6) together with a pore region between transmembrane helices S5 and S6. Both human Cav1.3 constructs used in expression studies (Cav1.3_L_ and Cav1.3_S_) contain the alternatively spliced exon 8A in domain I (Supplementary File: Figure S1). Alternative splicing in exon 43 located in the C-terminus results in a premature stop codon lacking the distal domain of the C-terminal modulator (CTM) (referred to as Cav1.3_S_, Supplementary File: Figure S1) and is highly expressed in the brain, but not in the heart [20]. Mutations related to cardiac conduction disturbances are indicated by a red dot. R930H is located extracellularly in the S1–S2 linker of repeat III. Mutations related to neurologic disorders as epileptic seizures, autism or developmental delay are indicated by a blue dot and mutations related to primary hyperaldosteronism by a black dot. The EF, pre-IQ and IQ motifs function as interaction sites for calmodulin. (**d**) Expression of human *CACNA1D* exon 22 (harboring the variant at residue 930) in different human heart compartments and the brain. A real-time PCR and 2^−ΔΔCt^-method was used to describe the relative *CACNA1D* mRNA expression in different heart tissue compartments and total brain normalized to atrial *CACNA1D* expression.

To identify the disease-associated variant which affects several family members with SAN disease and/or epileptic seizures (Figure 1a, Table 1), whole-exome sequencing was performed in four affected family members (I:2, II:2, III:1 and III:4). The statistics of sequencing quality are summarized in the Supplementary File: Table S1. Exome sequencing produced a mean of 53,533,464 total reads per sample, an average of 98.4% which could be successfully aligned to the reference genome, with a mean average sequencing depth on target of 80.9×. The mean fraction of the target that was covered with at least 20× was 92.8%. On average 335,421 variants were called per sample. Among them, about 13,450 variants per sample had a predicted consequence on protein sequence (splice region, insertion, deletion, non- synonymous single nucleotide variants (SNVs)). We focused on variants present in all four tested family members and identified 34 shared variants, which were additionally absent or very rare (MAF < 0.1%) in the gnomAD database. Of them, 14 variants (12 non-synonymous SNVs, one deletion, one splice site variant) were located in genes with a demonstrated cardiac expression and additionally predicted to affect protein function by more than 50% of the prediction algorithm integrated in VarCards database (Supplementary File: Table S2). From this candidate gene list, *CACNA1D* and *RNF207* were already described to be associated with inherited cardiac conditions (*CACNA1D*: sinoatrial node dysfunction [12]; *RNF207*: Long-QT Syndrome (LQTS) [27]). Of note, genetic variation in *CACNA1D* was also associated with epilepsy [25]. Since the LQTS phenotype was not present in the index family, we continued with a further characterization of the heterozygous variant NM_000720.3:c.2789G>A in the *CACNA1D* gene (Figure 2a) as a possible causative gene variant for the clinically combined phenotype of SND and epilepsy. This variant leads to a substitution of the hydrophilic and polar amino acid arginine to a histidine at residue 930 (p.Arg930His; shortly: R930H), which is located in the extracellular linker connecting helices S1 and S2 of domain III of the Cav1.3 α1-subunit (Figure 2c). Moreover, the wild-type residue arginine revealed a high degree of paralogous and orthologous conservation (Figure 2b). The altered allele was listed in the gnomAD database with a minor allele frequency (MAF) of 0.05%, predicted to affect protein function by 21 of 23 prediction tools (VarCards [28]; Damaging Score: 0.91, and the CADD score [29] also calculated the variant as one of the most harmful 0.1% variants in the whole genome (CADD-Phred: 34.0)).

To investigate the segregation of this variation with the disease in the family (Figure 1a), additional available family members were genotyped and clinically re-evaluated. In five clinically affected cases (four family members with SAN dysfunction or AVB, without documented signs of epilepsy, and one family member with documented epilepsy but no evidence for SND), the heterozygous variant was present, increasing the total amount of variant carriers in this family to nine. In the other five healthy family members, the mutant allele was absent. Therefore, the R930H variant in *CACNA1D* co-segregates in the family with the disease, however not all variant carriers show the combined clinical spectrum of cardiac and neurologic symptoms. To further investigate the presence of genetic variants in the *CACNA1D* gene in idiopathic forms of SND, we performed a complete genetic analysis of the coding regions of all exons in a cohort of 39 unrelated patients. Here, no additional putative pathogenic variants were detected, indicating that *CACNA1D* variants might be present in rare and complex (SND + epilepsy), but not isolated phenotypes of SND. 

### 2.3. Expression of CACNA1D in Human Cardiac Tissue

The human *CACNA1D* gene consists of 49 exons and, for some of these exons, tissue-specific alternative splicing has been demonstrated [20,30]. The expression pattern of *CACNA1D* in the mouse heart has been investigated in detail, however for the human heart little information is available concerning the expression level in the heart conduction system. As the identified nucleotide exchange leading to the p.R930H substitution was identified in exon 22 of *CACNA1D*, we first investigated the relative expression pattern of this particular exon in different heart compartments and in the brain (Figure 2d). Targeted transcripts of *CACNA1D* exon 22 were abundantly expressed in the atrium and nearly absent in the ventricle. With respect to the conduction system, expression of *CACNA1D* exon 22 was highest in the sinus node, followed by the AV node, whereas in Purkinje fibres, transcript levels were low. In contrast, expression of *CACNA1D* exon 22 was 8-fold higher in samples of the total human brain compared to the expression level in the atrium.

### 2.4. Cellular Localization of Transfected Human Cav1.3-R930H in HEK293 Cells

To analyze whether the p.R930H variant leads to an altered intracellular localization of the Cav1.3 channel (trafficking deficiency), HEK293T cells were transiently transfected with native and mutant *CACNA1D* (human long isoform), as well as the α_2_δ_1_ and ß_2_ accessory subunits. Localization of the wild-type and mutant Cav1.3 channel was determined by immunofluorescence staining. The wild-type as well as the R930H mutant Cav1.3 channels co-localized with the co-transfected plasma membrane marker. Quantification of the surface expression is difficult to obtain in single cell fluorescence experiments. However, from the cellular distribution of the fluorescence we determined there are no indications for a major mistrafficking of the R930H variant in Cav1.3_L_ (Supplementary File: Figure S2).

### 2.5. The CACNA1D R930H Variant Induces Isoform-Dependent Alterations in Cav1.3 Channel Activity and Channel Function

**Figure 3:**
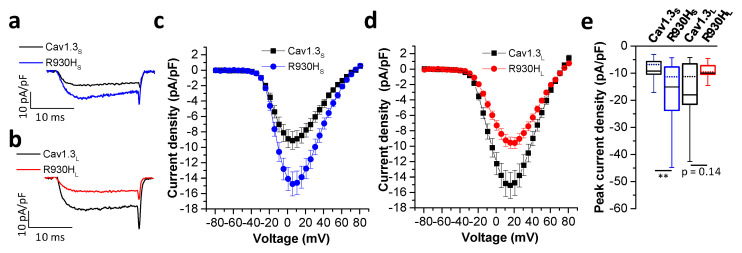
The R930H variant induces transcript variant dependent gating changes of Cav1.3 channels. (**a**) Representative current traces of wild-type Cav1.3_S_ and mutant Cav1.3_S_ R930H (R930H_S_) or (**b**) wild-type Cav1.3_L_ and mutant Cav1.3_L_ R930H (R930H_L_) upon a depolarization step to the potential of their maximal inward currents. (**c**,**d**) Current-voltage relationships of the *I*_Ca_ (mean ± S.E.M.) of (**c**) wild-type versus mutant short and (**d**) long Cav1.3 transcript variants. Note that the R930H variant enhanced current densities in the short splice variant. In contrast, current densities were reduced in the mutant long splice transcript variant. (**e**) Boxplots of the peak current densities obtained from depolarization to the respective voltage of the peak current (displayed as median (dotted lines), 25/75 percentile (whiskers) and mean (black lines)); median and 25/75 percentiles. In pA/pF: wild-type Cav1.3_S_: −6.8 ± (−10.5/−5.7), n = 32 and R930H_S_: −11.3 ± (−23.7/−7.7), n = 39, *p* = 0.0016; wild-type Cav1.3_L_: −11.2 ± (−19.1/−6.4), n = 55 and R930H_L_: −9.5 ± (−10.4/−7.2), n = 32, *p* = 0.14: **: *p* < 0.01. Statistics were performed using the Mann–Whitney test for both splice variants.

**Figure 4:**
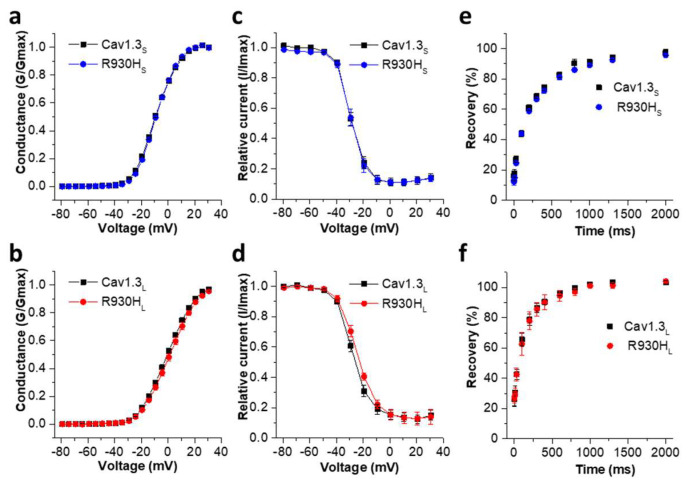
R930H does not alter activation and inactivation parameters of Cav1.3_S_ and Cav1.3_L_. (**a**) Voltage-dependence of activation of the wild-type and mutant Cav1.3_S_ and (**b**) Cav1.3_L_ variants. (**c**) Voltage-dependence of inactivation of wild-type Cav1.3_S_ versus R930H_S_ and (**d**) wild-type Cav1.3_L_ versus R930H_L_. (**e**) Recovery from inactivation of wild-type Cav1.3_S_ versus R930H_S_ (τ Cav1.3_S_: 270.1 ± 14.4 ms, n = 10; τ R930H_S_: 261.9 ± 18.2 ms, n = 12; not significant, unpaired Student’s *t*-test) and (**f**) wild-type Cav1.3_L_ versus R930H_L_ (τ Cav1.3_L_: 166.1 ± 20.56 ms, n = 5; τ R930H_L_: 180.6 ± 36.4 ms, n = 5; not significant, unpaired Student’s *t*-test. Consistent with the literature, recovery from inactivation was faster in the Cav1.3_L_ variant (τ Cav1.3_L_ vs. τ Cav1.3_S_ *p* = 0.0011, unpaired Student’s *t*-test) [20]. However, this parameter was not affected by the R930H variant. Recovery from inactivation was determined by 10 ms test pulses to V_max_ at different time points after a 5-s conditioning pulse to the potential of the peak currents followed by a test pulse, as described in the Materials and Methods section. Data were fitted using a mono-exponential function. Data in (**a**–**d**) are shown as mean ± S.E.M. For parameters and statistics see Table 2.

The R930H variant is located in the extracellular linker connecting the S1 and S2 transmembrane segment of domain III of the Cav1.3 channel (Figure 2c). Recently, it was reported that the S1–S2 linker is important for the gating of cation channels such as HCN [31] and TRPA1 [32]. Therefore, we hypothesized that R930H mutant channels might have altered channel gating properties, e.g., due to an impaired/altered voltage-to-gate sensing. To test this hypothesis, we characterized the properties of mutant Cav1.3 R930H channels in both the long and short human isoform (Cav1.3_S_ and Cav1.3_L_) (Supplementary File Figure S1 and Figure 2c), as Cav1.3_S_ is highly expressed in the brain, but not in the heart [20]. To this end, whole-cell patch clamp experiments were performed with tsA-201 cells transiently transfected with wild-type or mutant human Cav1.3_S_ or Cav1.3_L_ (Figure 3 and Figure 4).

Both transcript variants of Cav1.3 showed typical bell-shaped current voltage relationships (I/V curves) (Figure 3c,d). As previously reported [20], the I/V curves peaked at slightly more negative potentials in Cav1.3_S_ compared to Cav1.3_L_ channels (Figure 3b,d), reflecting the known negative shift in the voltage-dependence of activation (Table 2). The R930H variant significantly increased the current density of Cav1.3_S_ compared to the wild-type (Figure 3a,c). In contrast, this effect was absent when the variant was introduced into the Cav1.3_L_ channel variant. Instead, mutant Cav1.3_L_ current density was reduced (Figure 3a,d), although this change did not reach statistical significance due to a larger current amplitude variation in cells transiently expressing wild-type Cav1.3_L_ channels (Figure 3e). 

In contrast to current amplitudes, the variant did not affect the voltage of half-maximal *I*_Ca_ activation (Figure 4a,b) and inactivation (Figure 4c,d) (for statistics see Table 2). However, the variant significantly increased the steepness in the voltage dependence of activation compared to wild-type Cav1.3_S_ (Table 2). This indicates a stronger coupling of voltage sensing to pore opening once the activation threshold is reached. 

In addition, the long and short human R930H mutant channels appear to maintain their Ca^2+^ selectivity, as indicted by their unaltered reversal potentials (V_rev_) (Table 2). In accordance with previous findings [20], the time course of recovery from inactivation was significantly faster for the wild-type Cav1.3_L_ compared to Cav1.3_S_. However, in both channel variants, the recovery time course was not affected by the R930H variant (Figure 4e,f). 

We also measured the ON-gating charge (Q_ON_) for Cav1.3_L_ and R930H channels as a measure for changes in the channels’ surface expression and of channel open probability, which can be estimated as the ratio of Q_ON_ versus tail currents, as previously described [20]. The total Q_ON_ was not significantly different between the wild-type and mutant Cav1.3_L_ (Supplementary File: Figure S3a,b) supporting the fluorescence microscopy data which indicated normal channel trafficking (Supplementary File: Figure S2). We also detected no significant differences in Q_ON_/*I*_tail_ (Supplementary File: Figure S3c). Taken together, our data indicate that the reduced currents observed in the long Cav1.3 transcript are not caused by trafficking defects related to the presence of the R930H exchange, while on a functional site, the variant does not impair the efficiency of the opening of the Cav1.3_L_ variant by voltage. As previously described, the Q_ON_ of Cav1.3_S_ isoforms is too small for reliable quantification [20]. Thus, whether the increased currents of R930H in the short Cav1.3_S_ variant resulted from enhanced trafficking or altered gating currently remains unresolved. However, there are some indications that in the mutant Cav1.3_S_ channels (harboring R930H), an altered gating is present, since the voltage-dependence of the activation of mutant Cav1.3_S_ channels displayed a significantly increased steepness in the voltage-dependence of activation compared to Cav1.3_S_ wild-type channels (Table 2). 

As the R930H variant introduced no or only a minor loss-of-function in human Cav1.3_L_ channels, we also studied the R930H variant in a long rCav1.3_L_ variant, which differs in alternative splicing from the human construct employed in this study (Supplementary File: Figure S1). Here, we also found a loss-of-function due to a gating effect, albeit with a different mechanism (Supplementary File: Figures S4 and S5). This long transcript variant cloned from rat lacks cassette exon 44 (in the long C-terminus) and utilizes the mutual exclusively spliced exon 8B (Supplementary File: Figure S1). When introduced into rCav1.3_L_, the current densities and the voltage-dependence of activation were not altered (Supplementary File: Figure S4a–d). However, a loss-of-function was observed for the R930H variant by altered inactivation gating, with a small but significant negative shift in the voltage-dependence of inactivation (Supplementary File: Figure S4e,f) and accelerated kinetics of inactivation (Supplementary File: Figure S5), caused by significantly accelerating the slow phase of inactivation (Supplementary File: Figure S5). Both changes are compatible with a loss of rCav1.3_L_ channel function. 

Previous studies have shown that divalent cations, such as Cu^2+^ or Zn^2+^, can inhibit Cav2.3 and Cav3.2 calcium channels by interacting with extracellular binding sites, also formed by histidine residues localized in the IS1-S2-linker, as in the R930H mutant [33,34,35]. We therefore tested the possibility that introduction of an additional histidine on the extracellular surface of R930H channels would enhance the sensitivity for Zn^2+^. Cav1.3 transfected cells were superfused with 30 µM Zn^2+^, corresponding to the previously described free Zn^2+^ concentration in the brain [36]. Application of Zn^2+^ resulted in a comparable reduction of *I*_Ca_ amplitude in both wild-type and R930H mutant Cav1.3_S_ channels, which was partially reversible upon washout with a standard bath solution (Supplementary File: Figure S6a,b). The normalized current–voltage relationships measured before, during and after (wash out) Zn^2+^ application revealed no difference in the voltage-dependence of activation between wild-type and mutant Cav1.3_S_ R930H (Supplementary File: Figure S6c).

## 3. Discussion

In this study, we performed whole-exome sequencing (WES) to identify the causative gene mutation in a family with SND, variably characterized by sinus arrest, sinus bradycardia and low heart rates due to high AV blockade. Interestingly, four of the nine variant carriers were affected by an additional neurologic phenotype encompassing epileptic seizures and learning disabilities, mainly ADHD. A rare heterozygous missense variant (c.2789G>A; p.Arg930His) in *CACNA1D* was identified and co-segregated with the affected family members. So far, mutations in *CACNA1D* encoding the α_1_-subunit of Cav1.3 L-type Ca^2+^ channels, are quite rare and have been described in individuals with SND (p.Gly403dup, p.Ala376Val). However, in these cases the bradycardic phenotype is part of a syndromic disease additionally presenting with congenital deafness (SANDD); of note, only homozygous carriers of the identified gene mutation are clinically affected [12,22]. Neurologic symptoms, seizures in particular, were not reported in the SANDD patients. In addition to the cardiac phenotype observed in SANDD, heterozygous *CACNA1D* mutations also cause high risk for a broad neurodevelopmental disease spectrum, including childhood epilepsy, autism spectrum disorders (A769G, G407R, V401L, Q567H, V1482L) [24,25,37,38], attention deficit hyperactivity disorder-like symptoms (S672L) [23], as well as primary hyperaldosteronism (I770M, G403D, A769T, V259D) [26,39,40] and congenital hypoglycemic hyperinsulinemia (G403D) [41]. Functional analysis of these de novo mutations suggested a gain-of-function mechanism leading to the observed phenotypes [23,25]. However, in three individuals affected by mutations G403D (2 individuals) [26,41] and V259D [39], cardiac abnormalities were also present, including bradycardia and AVB. Garza-Lopez and co-workers reported changes of voltage-dependent inactivation of Cav1.3 by the *CACNA1D* mutation Q567H. A homozygous carrier of this mutation presented with epilepsy and developmental delay, which was also accompanied by moderate hearing impairment [37], suggesting a partial phenotypic overlap between *CACNA1D* variants with either gain-or-loss of function phenotypes. In line with this, the clinically affected family members with the R930H variant identified in this study also showed aspects of the SANDD phenotype (e.g., bradycardia) in addition to epilepsy. In contrast to the known *CACNA1D* mutations, which are mainly de novo or recessive, the R930H variant is inherited in an autosomal dominant manner with a variable penetrance of clinical features, i.e., some variant carriers reveal the combined phenotype of epilepsy and SND or impaired cardiac conduction, whereas others only show cardiac signs or epileptic seizures. For the SANDD phenotype mutations G403dup and A376V, as well as mutation Q567H in Cav1.3, a recessive inheritance has been described and thereby only biallelic mutation carries were affected by deafness or severe hearing impairment [12,22,37]. This is in line with the absence of deafness in all heterozygous *CACNA1D* R930H variant carriers. With respect to the severity of the cardiac phenotype and the occurrence of deafness, the phenotypic expression of *CACNA1D* mutations resembles the phenotypic spectrum seen in loss-of-function mutations in the *KCNQ1* gene encoding the α_1_-subunit of the cardiac potassium channel Kv7.1. Here heterozygous mutations result in Romano–Ward Syndrome, a cardiac ventricular repolarization abnormality which is characterized by a prolonged QT interval and a propensity for ventricular tachycardia [42]. Biallelic mutation carriers develop a more severe form of LQTS (Jervell and Lange-Nielsen Syndrome) with a higher incidence of sudden death and are additionally affected by deafness [43]. We demonstrated that exon 22 encompassing arginine 930 is expressed in cardiac conduction tissue as well as in the brain, thus the p.R930H variant must result in dysfunctional Cav1.3 channels in the heart as well as in neurons, which is in line with the combined phenotype of epileptic seizures and bradycardia seen in R930H variant carriers.

We provide novel, relatively strong evidence for the pathogenicity of the R930H variant due to the co-segregation of the phenotype with the variant in this family, a phenotype compatible with Cav1.3 dysfunction and the presence of variant-induced functional changes. However, the functional changes observed in our electrophysiological studies are much less pronounced as previously described in SANDD (strong loss-of-function) or in individuals with neurodevelopmental symptoms (pronounced gating changes permitting gain-of-function) [44]. However, as previously discussed [44], the interpretation of functional changes in terms of gain or loss of channel function is complex. While gating changes, such as slowing of the inactivation time course or the shift of activation voltage to more negative potentials, support enhanced channel activity, parallel changes, such as reduced maximal current density (see e.g., G407R mutation in ASD) or negative shifts of steady-state inactivation voltage (as in many type-2 mutations [44]), support channel loss-of-function. Therefore, the net effect must depend on the electrical activity pattern(s) and may therefore differ between tissues and cells. The R930H variant described here and the mutation Q567H (which, interestingly, are both located in extracellular S1–S2 linkers and introduce histidine) show more complex phenotypes which, based on our current understanding of genotype–phenotype relationships, can be explained by either gain (R930H: seizures, learning disability; Q567H: seizures, ASD, hyperactivity) or loss-of channel function (R930H: SAN dysfunction; Q567H: hearing impairment). Notably, both mutations show both gain and loss of function features in electrophysiological studies. The R930H variant results in a gain-of-function when present in the Ca_v_1.3_S_ variant and loss-of-function features when introduced into the Cav1.3_L_ variant of humans or rats. Therefore, the loss-of-function phenotype may predominate in the heart where mostly Cav1.3_L_ variants are expressed [20]. In contrast, Cav1.3_S_ variants comprise up to half of the Cav1.3 α_1_-subunit species in certain brain areas [20], allowing a gain-of-function phenotype resulting from the enhanced Cav1.3_S_ activity to predominate. Likewise, the reduction of current density induced by the Q567H mutation in the homozygous proband can nicely explain this hearing impairment, whereas the slowing of inactivation could enhance Cav1.3 currents in neurons [37].

Our findings indicate that the R930H variant alters voltage-dependent gating of the Cav1.3 ion channel complexes in native cardiac and neuronal tissue. However, one limitation is that we have chosen to perform electrophysiological recordings in 15 mM extracellular calcium. However, this extraordinary high divalent cation concentration is expected to significantly alter most voltage-dependent channel gating and could have obscured the effects of the variant. Thus, future studies quantifying the effects of the R930H variant in more detail should consider using a more physiological calcium concentration.

The exon 22, in which we identified the p.R930H exchange, shows a much higher transcriptional expression in the brain compared to cardiac tissue, which may raise the possibility that the sinus node phenotype that we have observed in our patients might, in part, be secondary and results from an altered autonomic drive of the SAN, as opposed to the intrinsic calcium channel-mediated automaticity. This is of particular interest since a recent study suggests an additional and novel role of Cav1.3 channels in initiating and maintaining automaticity in dormant sinoatrial node cells upon β-adrenergic stimulation [45]. Whether these effects are altered on a neuronal and/or sinoatrial level by the Cav1.3 R930H variant currently remains an open question.

Four siblings with the R930H variant (III:1–III:4) were diagnosed and treated for ADHD. Substances used for ADHD treatment, such as methylphenidate, increase the heart rate due to their indirect sympathomimetic effects by releasing norepinephrine from intraneuronal stores of adrenergic neurons and inhibiting reuptake. This could mask or compensate for the bradycardia by the mutant Cav1.3 channel in the sinus node and might finally explain the lower penetrance of the cardiac phenotype in this particular family member without obvious signs of SND.

For now, we can only speculate why R930H appears with 1:4000 in control populations (i.e., gnomAD). Other genetic or epigenetic factors might be relevant to influence the penetrance of the Cav1.3 R930H exchange, such as the ADHD medication discussed above. The R930H variant clearly affects the function of the Cav1.3 channel in a splice form specific manner and the mutation co-segregates with the phenotype in the patients we report here. Despite that, the R930H variant does not induce a purely monogenetic disorder evident by a full or perfect penetrance, and the association with epilepsy and sinus node dysfunction we report here should be taken seriously.

## 4. Materials and Methods

### 4.1. Study Population

All 32 probands with idiopathic SND included in the present study gave written informed consent for genotyping in accordance with the last Declaration of Helsinki and with recommendations by the local ethics committee before genetic and clinical investigation. Detailed clinical data, including cardiac symptoms, baseline and Holter ECGs and evaluation of clinical reports were obtained. Digitalized 12-lead ECG recordings with standard lead positions were measured in a graphic program for accurate measurements of PQ, QRS and QT, as well as RR intervals in three consecutive beats. ECG and cardiac rhythm analysis were independently performed by two cardiologists. In addition, members of the family with the heterozygous non-synonymous *CACNA1D* variant (pArg930His; shortly: R930H) were neurologically evaluated; the four siblings (III:1–III:4), who suffered from epilepsy were clinically examined by a child neurologist. Diagnostic work-up included a cerebral MRI scan, neurologic examinations and regular electroencephalogram (EEG) recordings (two to four times/year). They were followed up for 11 to 16 years. In addition, three apparently unaffected family members (II:2, II:3 and II:7) were also examined by EEG.

### 4.2. Whole-Exome Sequencing 

Whole-exome sequencing was performed on four affected family members (I:2, II:2, III:1 and III:4) at Novogene (Beijing, China) using the Agilent SureSelect Human All Exon kit (Agilent Technologies, Santa Clara, CA, USA) to prepare the sequencing libraries and the ‘Illumina HighSeq 2000′ platform for high-throughput sequencing. The Burrow-Wheeler Aligner (BWA) [46] was utilized to map the paired-end clean reads to the human reference genome (hg19). SAMtools [47] was used for sorting the BAM files and PICARD was used to mark duplicate reads. GATK v3.8 [48] was used to detect SNVs and Indels and ANNOVAR was used to annotate the called variants. We first filtered for rare, non-synonymous coding variants (MAF < 0.1%, based on the gnomAD variant database “https://gnomad.broadinstitute.org/ (accessed on 1 February 2022)”) with a possible impact on protein function (missense, stop-gained or stop lost, predicted effect on mRNA splicing, deletions and insertions). As pedigree analysis was suggestive for an autosomal dominant inheritance, variants being present in all four affected family members were filtered first and then investigated for potential pathophysiologic links between the underlying gene and cardiovascular disease, using the OMIM, Human Phenotype Ontology (HPO) and PubMed databases. The RPKM values of the HPA RNA-seq normal tissue database [49] were used to evaluate the relative expression and relevance of the candidate genes in cardiac tissue: RPKM values > 0.1 were considered to resemble cardiac expression. For in silico assessment of variant pathogenicity, VarCards, an integrated genetic and clinical database for coding variants in the human genome (“http://varcards.biols.ac.cn/ assessed on 1 February 2022”), was used to predict potentially pathogenic effects of non-synonymous SNVs on protein function. Variants with a high damaging score (ratio of programs with a deleterious result/total number of programs >0.5) were further selected. All available DNA samples were further analyzed by targeted Sanger re-sequencing for co-segregation of a variant of interest with the clinical phenotypes in the family. Classification of identified variants was performed according to the ACMG criteria [50], with the notion that the majority of genes might not be linked to the phenotype so far (gene of unknown pathophysiological significance).

### 4.3. Screening for Genetic Variants in the CACNA1D Gene in 39 Additional SND Patients

After isolation of genomic DNA from peripheral blood lymphocytes of the probands, all 49 exons and >20 bp of flanking intronic sequences of the human *CACNA1D* gene (long form) were amplified by PCR and analyzed by direct Sanger sequencing using standard procedures. The obtained nucleotide sequences were compared with wild-type *CACNA1D* cDNA sequences (NM_001128840.1, NP_001122312.1 containing the exon 8A; NM_000720.2, NP_000711.1 containing the alternative exon 8B, respectively) (Supplementary File: Figure S1). Primer sequences are available upon request.

### 4.4. Cardiac-Specific Expression of CACNA1D

The total RNA isolated from the human atrium, ventricle, SAN, AV node and Purkinje fibres was purchased from Analytical Biological Services (ABS) Inc (Wilmington, DE, USA). The tissue samples from the atrium, ventricle, SAN and Purkinje fibres were collected four days post-mortem from a 41-year-old Caucasian female who died due to liver failure. In addition, AV node samples were obtained from a 61-year-old male Caucasian who suffered from asthma and chronic obstructive pulmonary disease (COPD). For both donors, no cardiovascular, kidney/urinary, neurological or digestive disorders were reported. ABS Inc. states that the post-mortem collections were accredited by the government and that a donor’s consent was obtained before removing any tissues for research. The total RNA obtained from a human brain of a single healthy donor was purchased from ZYAGEN (San Diego, CA, USA). RNA integrity of all samples was checked on the Agilent 2100 Bioanalyzer system using the RNA Analysis Kit (Agilent, Santa Clara, CA, USA). 

An amount of 2 µg of total RNA from each sample was reverse transcribed using the QuantiTect Reverse Transcription Kit (Qiagen, Hilden, Germany) in a 40 µL reaction according to the manufacturer´s instructions. qPCR was performed on 1 µL aliquots of at least three different cDNA samples in triplicate using Rotor-Gene SYBR Green PCR Kit and Rotor-Gene Q (Qiagen, Hilden, Germany) in 25 µL reactions. All qPCR reactions were performed using a 5 min initial activation step at 95 °C, followed by 45 cycles (15 s at 95 °C, 30 s at 65 °C and 30 s at 72 °C). PCR primers were exon spanning to avoid amplification of the genomic template. PCR was performed to detect *CACNA1D* transcripts containing exon 22 (containing the mutant residue at position 930) with forward primer 5′-TAGCAAGACCAACCCGATCCGCGTAG-3′ and reverse primer 5′-GTAACCCAGTATCGTGTTCCGGAAGGAG-3′ (amplicon length: 165 bp). The efficiency of the primer set was tested to be and found to be similar to the amplification efficiency of GAPDH. Ct-values were determined, normalized to GAPDH and averaged. The 2^−Δct^ values were used to describe relative mRNA expression. To compare the relative amount of expression of a target gene in a given tissue with the expression in the atrium, the 2^−ΔΔct^-method was used.

### 4.5. Site-Directed Mutagenesis of CACNA1D Constructs

Human *CACNA1D* constructs: Human wild-type Cav1.3α_1_-subunits (reference sequence EU363339) containing exon 8a and either (a) the whole C-terminus (long isoform 1; Cav1.3_L_) or (b) the alternatively C-terminally spliced exon 43 (short isoform 1; Cav1.3_S_) were cloned into the pGFP^minus^ vector as previously described [17,20]. The short isoform lacked parts of the *CACNA1D* C-terminus due to the use of an alternative splice site in exon 43 (Supplementary File: Figure S1). The mutant variant R930H was introduced into both Cav1.3 splice variants using standard polymerase chain reaction approaches and hereafter verified by Sanger sequencing (Eurofins Genomics, Ebersberg, Germany).

Rat *CACNA1D* constructs: A 7.9 kb NotI fragment of rat *CACNA1D* subunit cDNA (GenBank: D38101.1) was inserted into the mammalian expression vector pCMV6b. This expression construct was kindly provided by Dr. S. Seino (Kobe, Japan). After digestion with NotI, the resulting fragment was subcloned into pAlter-Ex1. The pAlter-Ex1 vector containing r*CACNA1D* cDNA was used for site-directed mutagenesis with the Altered sites II in vitro Mutagenesis System (Promega, Madison, WI, USA). For the generation of r*CACNA1D* R969H mutation (corresponding to R930H in h*CACNA1D*), a 33-mer antisense oligonucleotide including the mismatched base (Arg969: CGC → His: CAC) was synthesized. The mutant r*CACNA1D* cDNA was confirmed by sequencing (GATC GmbH, Konstanz, Germany). The rat *CACNA1D* construct corresponds to the long human Cav1.3_L_ isoform, but utilizes the mutual exclusively spliced exon 8b and, in addition, contains exon 44.

### 4.6. Immunofluorescence Staining of Cav1.3 R930H Transfected HEK 293T Cells

Cultured HEK 293T cells, cultivated in DMEM supplemented with 2 mM L-Glutamine, 100 U/10 µg/mL Penicillin/Streptomycin and 10% FCS at 37 °C and 5% CO_2_, were seeded on poly-L-Lysine coated 8-well glass CultureSlides and transfected with 1 µg plasmid DNA (WT and mutant Cav1.3 (long isoform), together with the subunits Cavβ_2_ and Cav α_2_δ_1long_ at a ratio of 4:1:1 and pDsRed-Monomer-F Hyg- (plasma membrane staining) (Clontech, San Jose, CA, USA) using FuGENE 6 Transfection Reagent (Roche, Grenzach-Whylen, Germany), according to manufacturer’s instructions. Forty-eight hours after transfection, HEK 293T cells were incubated with a microtubuli stabilizing buffer (1x MTSB) for 5 min to maintain the architecture of the cytoskeleton. After fixing the cells with 4% PFA for 20 min, they were permeabilized with 0.1% Triton X-100 for 5 min and blocked with 5% BSA for at least 1 h. A primary goat anti-Cav1.3-antibody (1:50 in 5% BSA, Santa Cruz, Dallas, TX, USA) was incubated overnight at 4 °C. After three washing steps (1x PBS), secondary Alexa Fluor 488-labeled rabbit anti-goat IgG (1:250 in 5% BSA, Life Technologies, Darmstadt, Germany) was incubated for 1 h before mounting with ProLong Gold Antifade Reagent (Life Technologies, Darmstadt, Germany). Confocal microscopy was performed with an LSM 510 Meta and a 63x/1.4 oil lens (Zeiss, Oberkochen, Germany) and the obtained images were modified using an LSM Image Browser (Zeiss, Oberkochen, Germany). The same settings for image acquisition and processing were applied across all conditions.

### 4.7. Patch-Clamp Recordings of Wild-Type and Mutant Human Cav1.3 Transcripts (Cav1.3_S_ and Cav1.3_L_) in tsA-201 Cells

The tsA-201 cells were cultured in 10 cm dishes and transiently transfected with complementary DNA encoding wild-type or mutant human Cav1.3 α_1_-subunits (3 µg) together with auxiliary β_3_- (NM_012828) (2 µg) and α_2_δ-1- (NM_001082276) (2.5 µg) subunits and GFP (2 µg). β_3_ and β_4_ subunits are the most abundant beta subunits associated with voltage-gated calcium channels, including L-type channels, in the brain. Like β_1_ and most of the β_2_ subunit splice variants, they stabilize fast voltage-dependent inactivation kinetics and are therefore representative for the vast majority of the calcium channel associated beta subunits. Likewise, α_2_δ-1 is the most prominent isoform in the brain. Twenty-four hours after transfection, the cells were seeded onto 3 cm dishes coated with polylysine and subsequently kept at 30 °C/5% CO_2_. Electrophysiological recordings were performed 48–72 h after transfection.

Whole-cell patch-clamp recordings were performed at room temperature with an Axopatch 200B amplifier (Axon instruments, San Jose, CA, USA) using electrodes pulled from borosilicate capillaries with a resistance of 1.5–3.5 MΩ. All recordings were digitized at 50 kHz (Digitizer 1322A, Axon instruments, San Jose, CA, USA); low-pass filtered at 2 or 5 kHz, analyzed with pClamp 10.2 software (Axon instruments, San Jose, CA, USA) and compensated for 60–70% of the series resistance. The extracellular solution contained (in mM): standard bath: 15 CaCl_2_, 10 HEPES, 150 choline-Cl and 1 MgCl_2_ (adjusted to pH 7.4 with CsOH); bath for Zn^2+^ experiments: 15 CaCl_2_, 10 HEPES, 150 choline-Cl, 1 MgCl_2;_ and 0.03 ZnCl_2_ (adjusted to pH 7.4 with CsOH). The intracellular solution contained (in mM): 135 CsCl, 10 HEPES, 10 Cs-EGTA, 1 MgCl_2_, and 4 Na_2_ATP (adjusted to pH 7.4 with CsOH). 

To determine the current voltage (*I*/*V*) relationship, the cells were held at −80 mV and 20 ms square pulses and different voltages were applied. The voltage dependence of Ca^2+^ conductance was fitted according to a Boltzmann’s distribution. Steady-state inactivation was measured by applying a control test pulse (20 ms to the voltage of maximal inward current, Vmax) followed by 5-s conditioning steps to various potentials and a subsequent 20-ms test pulse to Vmax (30-s recovery between protocols). Inactivation was calculated as the ratio between the current amplitudes of the test versus control pulse. Steady-state inactivation parameters were obtained by fitting the data to a Boltzmann equation. Recovery from inactivation was determined by 10 ms test pulses to Vmax at different time points after a 5-s conditioning pulse to Vmax followed by a test pulse. 

For Zn^2+^ experiments, cells were perfused by an air pressure-driven perfusion system (BPS-8 Valve Control System, ALA Scientific Instruments, Farmingdale, NY, USA, flow rate: 250 µL/min). A total of 100 ms depolarizing stimuli to Vmax were applied at 0.2 Hz and IVs were recorded before, during and after (washout) micro-perfusion with Zn^2+^. The washout was performed by perfusion with a standard bath solution (15 mM Ca^2+^). 

The recordings were corrected for a junction potential of −9.3 mV (15 mM CaCl_2_) as previously described [51] and leak subtraction was performed either offline (steady-state inactivation and recovery from inactivation) or online using the P/4 protocol. The data were analyzed using Clampfit 10.2 (Axon Instruments, San Jose, CA, USA), Sigma Plot 12 (Systat Software Inc., Düsseldorf, Germany) or Graph Pad Prism 5.1 software (GraphPad Software Inc., San Diego, CA, USA).

### 4.8. Patch-Clamp Recordings of Rat Cav1.3 Transcripts (Corresponds to the Long Human Cav1.3_L_ Isoform) in CHO Cells

The cells were grown on 35 mm dishes (Nunc) to a confluency of about 50%. The cells in each dish were transfected with 4 µg of wild-type rCav1.3 or mutant rCav1.3 R930H cDNA in pCMV6b, 1 µg α_2_δ_1_ (Supplementary File: Figure S1) in pCDNA3.1, 1 µg β2b in pCDNA3.1 and 0.2 µg of pEGFP vector using Jetprime (peqlab, Erlangen, Germany), according to the instructions of the manufacturer. After 48 h, CHO cells were recorded in whole cell configuration at room temperature (22 °C) with an EPC-10 amplifier (HEKA). All recordings were digitized at 10 kHz, low-pass filtered at 2 or 5 kHz, analyzed with PulseFIT software (HEKA) and compensated for 60–70% of the series resistance. The pipettes had a tip resistance of 2.5–4.0 MΩ when filled with a solution containing (in mM): Cs-methane sulfonate 120, CaCl_2_ 5, MgCl_2_ 2, EGTA 10, MgATP 2 and HEPES 10 (pH 7.4 CsOH), yielding a [Ca^2+^_i_] of about 110 nM (calculated with WinMAxc). The cells were bathed in a solution containing (in mM): NMDG 130, CaCl_2_ 15, KCl 5 and HEPES 10 (pH 7.4, HCl).

### 4.9. Statistics

The data were tested for normality using a Shapiro–Wilk test. Statistical significance was determined by an unpaired Student’s *t*-test, a Mann–Whitney test and a two-way ANOVA followed by Bonferroni post-test, respectively. The data is presented as mean ± S.E.M. for the indicated number of experiments (n), unless stated otherwise. Statistical significance was set at *p* < 0.05.

## Figures and Tables

**Table 1:** Neurological and cardiac phenotypes in a family with a heterozygous *CACNA1D* variant, p.Arg930His (+).

ID	Sex	*CACNA1D* Variant	Baseline ECG HR [/min]; PQ; QRS; QTc [ms]	Holter ECG	Cardiac Phenotype	Neurologic Phenotype	EEG
I:1	m	−	75; 148; 100; 400	n.a.	LAHB		n.a.
I:2	f	+	52; 175; 92; 387	AVB I°; 42–109/min	LAHB		n.a.
II:1	m	−	85; 155; 82; 384	n.a.	−		n.a.
II:2	f	+	85; 126; 87; 379	SND, 40/min; pauses of 2.3 s 48–126/min	TTE: AVB III°; cardiac MRI unremark.		n.a.
II:3	m	+	56; 135; 73; 341	sinus arrhythmia; 48–103/min	−		n.a.
II:6	f	+	75; 117; 58; 398	sinus arrhythmia, 40–143/min	TTE unremark.		n.a.
II:7	f	+	70; 135; 89; 390	AVB I°, AVB II°, sinus arrhythmia; 47–152/min	−		n.a.
III:1	f	+	n.a. ^#^	SA	cardiac syncope, PM (3 y) *	IFE; ADHD	focal sharp waves: left occipital, left frontal and frontopolar
III:2	f	+	76; 127; 76; 354	sinus arrhythmia, AVB I°/II°; 70–154/min	−	IFE; ADHD	focal sharp waves: frontal and central
III:3	m	+	142; 94; 71; 400	unremarkable; 110–189/min	−	IFE; ADHD	focal sharp waves: left occipital
III:4	f	+	86; 157; 63; 387	AVB I°, AVB II°, SA; 64–142/min		IFE; ADHD	unremarkable

m, male; f, female; *, age of PM implantation; AVB, atrio-ventricular block; LAHB, left anterior hemi-block; SND, sinus node dysfunction; SA, sinus arrest; TTE, transthoracic echocardiography; ^#^ due to PM implantation; PM, pacemaker; ADHD, attention deficit hyperactivity disorder; IFE, idiopathic focal epilepsy; n.a., not available; +/−, presence in the heterozygous state; −/−, absence of variant, wild-type.

**Table 2:** Comparison of activation and inactivation parameters of wild-type (native) Cav1.3 and the Cav1.3 R930H channels.

	Activation				Inactivation		
	V_rev_ [mV]	Slope [mV]	V_0.5_ [mV]	n	V_0.5_ [mV]	Slope [mV]	n
**Cav1.3_S_**	67.62 ± 1.05	7.66 ± 0.11	−8.94 ± 0.41	50	−29.69 ± 1.06	4.45 ± 0.16	15
**R930H_S_**	67.63 ± 1.27	7.17 ± 0.10 **	−8.67 ± 0.43	39	−30.24 ± 1.00	4.47 ± 0.23	9
**Cav1.3_L_**	69.35 ± 0.65	9.24 ± 0.11	−0.14 ± 0.56	79	−25.72 ± 2.08	5.56 ± 0.23	18
**R930H_L_**	68.80 ± 0.82	9.24 ± 0.16	1.73 ± 1.06	32	−24.11 ± 1.12	6.22 ± 0.35	12

Activation and inactivation parameters were obtained after fitting normalized G/V relationships or normalized steady-state inactivation curves, as described in the methods section. Data are shown as mean ± S.E.M. from more than three independent transfections. ** = *p* < 0.01 versus Cav1.3_S_ using unpaired Student’s *t*-test. Long and short splice variants were analyzed separately. V_0.5_, voltage of half-maximal activation/inactivation; V_rev_, reversal potential; n, number of experiments. For composition of Cav1.3 transcripts, see Section 4.

## Data Availability

Not applicable.

## Supplementary Materials

The following supporting information can be downloaded at: https://www.mdpi.com/article/10.3390/ijms232214215/s1.
