# Supplementary material for: Whole Exome Sequencing Identifies a Heterozygous Variant in the Cav1.3 Gene CACNA1D Associated with Familial Sinus Node Dysfunction and Focal Idiopathic Epilepsy
Source: Int J Mol Sci. 2022 Nov 17;23(22):14215. doi: 10.3390/ijms232214215 (PMC9693521; doi:10.3390/ijms232214215)
Supplement: Supplementary file 1 [file ijms-23-14215-s001.zip › ijms-1958047-supplementary.pdf]

Figure S1

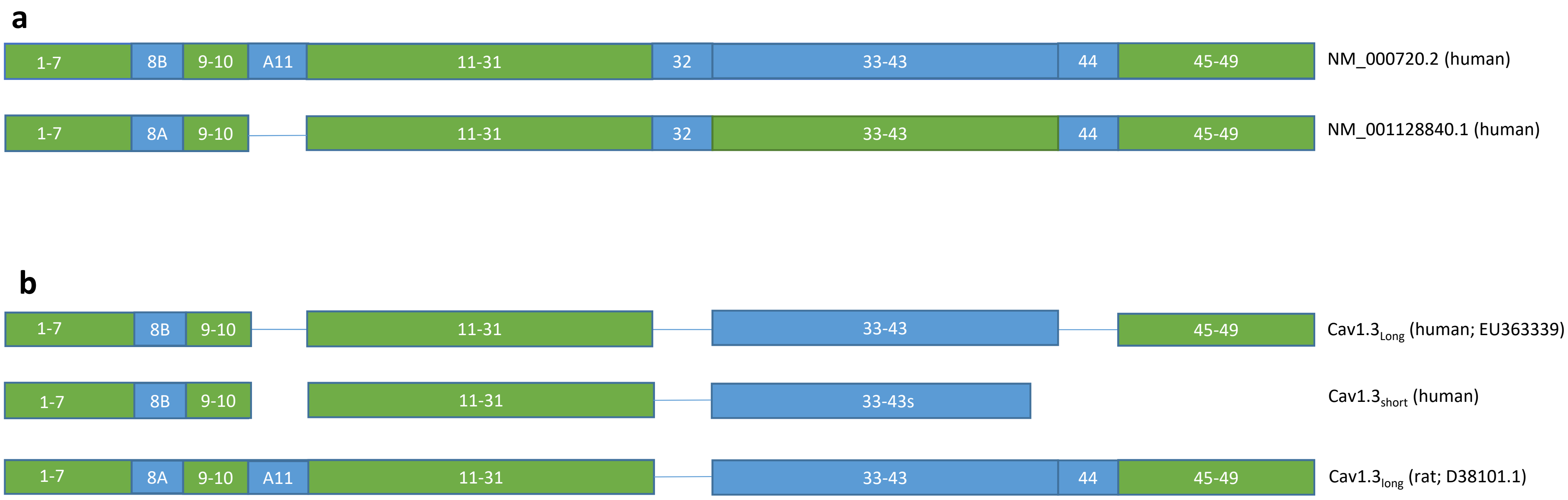

Figure S1. *Schematic drawing of the human and rat CACNA1D isoforms used.* (**a**) Isoforms used as reference sequence for sequence analysis of *CACNA1D* in SND patients (NM_000720.2 and NM_001128840.1) and (**b**) used as template cDNA for cloning of mutant and wild-type expression constructs (EU363339= Cav1.3_{long}, D38101.1=rat Cav1.3_{long}). Exons present in all isoforms are indicated in green. The short Cav1.3 α1 isoform used for functional analysis uses an alternative splice site in exon 43 encoding a C-terminally truncated Cav1.3 protein.

Figure S2

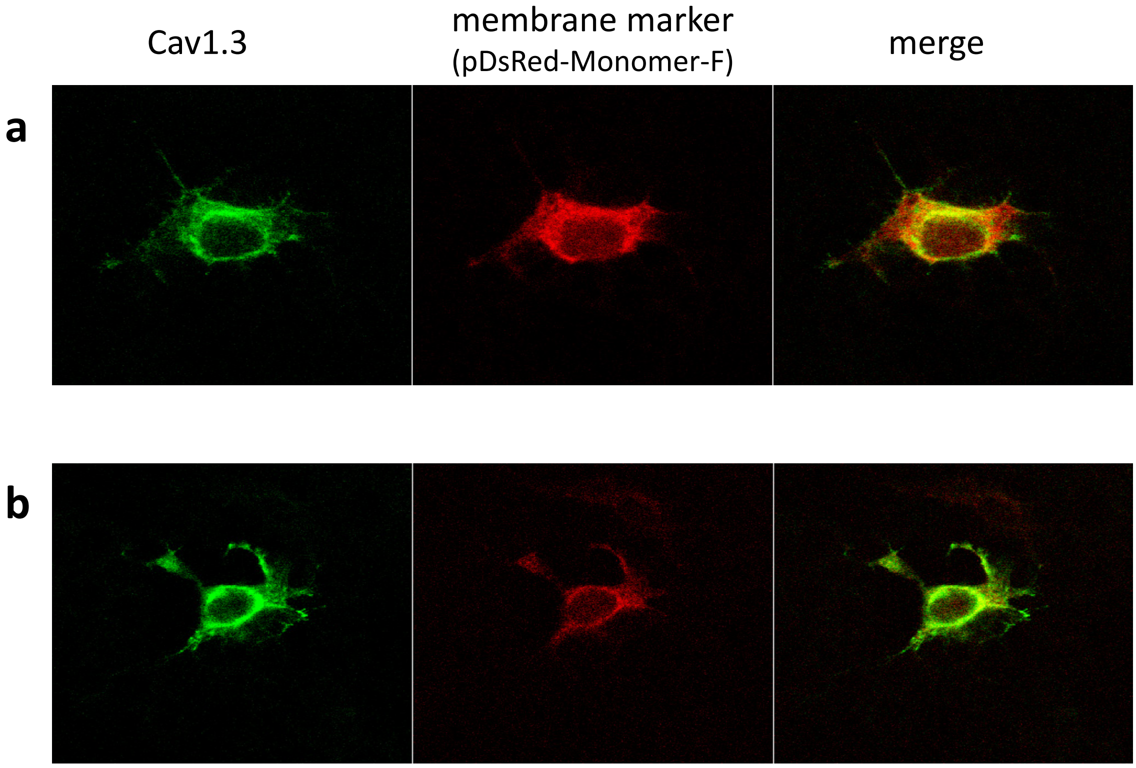

Figure S2. *Cellular localization of mutant Cav1.3$_L$ R930H.* (**a**) HEK293T cells were transiently transfected with WT and (**b**) mutant Cav1.3 and the accessory subunits Cav ß$_2$ and Cav $\alpha_2\delta_1$. Cav1.3$_L$ proteins are labeled in green and the co-transfected membrane marker (pDsRed-Monomer-F) is labeled in red.

Figure S3

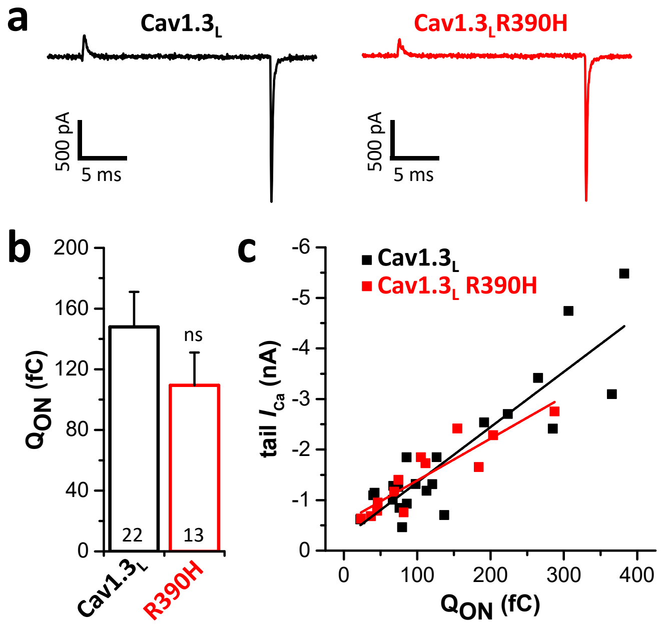

Figure S3. *Coupling of ON-gating current to the opening of Cav_L and Cav_LR390H.* (**a**) ON-gating currents were measured stepping from a holding potential of -80 mV to the reversal potential of each cell. Tail currents were induced by a repolarisation step to -80 mV. Illustrated are representative Q_{ON} and tail current traces for Cav1.3_L (*black*) and Cav1.3_L R390H (*red*). (**b**) Quantification of the integral of the ON-gating current for Cav1.3_L and Cav1.3_LR390H revealed a non-significant reduction of Q_{ON} for R930H. Numbers of cells analyzed are illustrated within the bar graph. Data are provided as mean ± S.E.M.. (**c**) Correlation of Q_{ON} and the peak tail current amplitude (tail I_{Ca}).

Figure S4

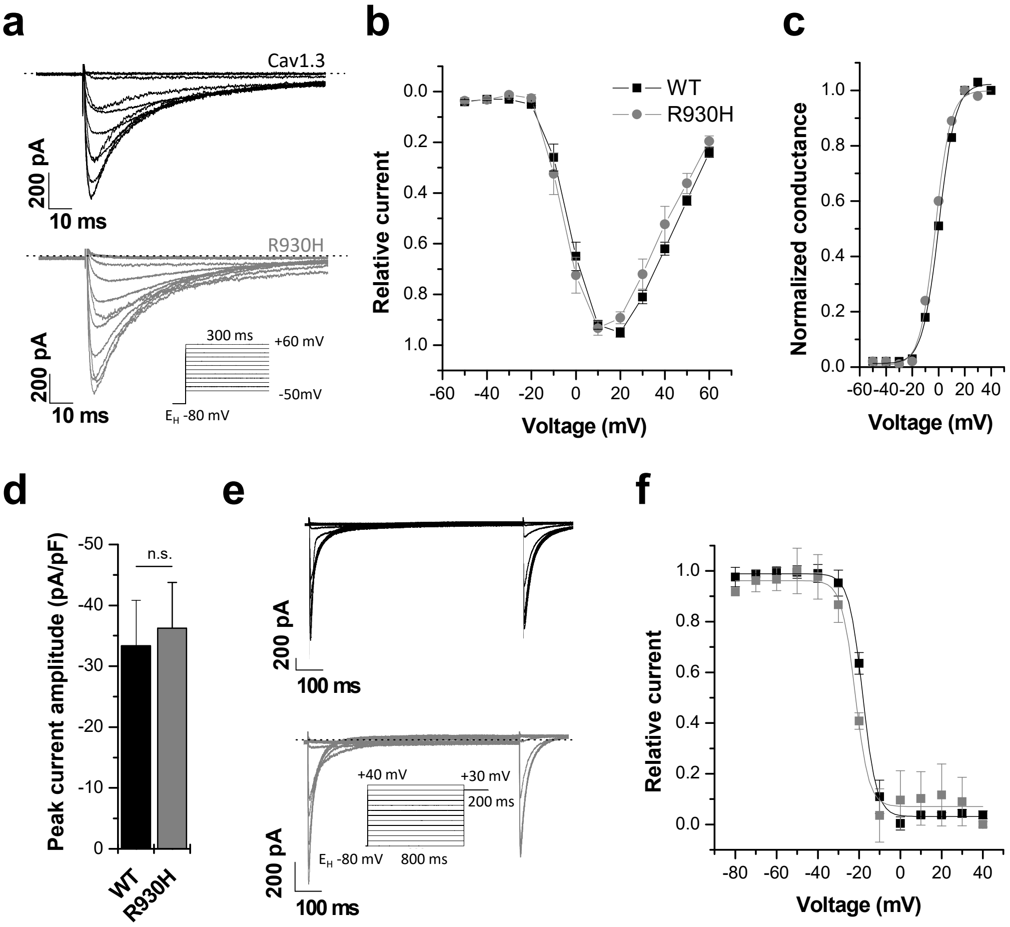

Figure S4. *Functional characterization of the rCav1.3$_L$ R930H mutation of the rat Cav1.3 channel.* (**a**) Whole-cell patch clamp recordings of wild-type rCav1.3$_L$ and the respective mutant rCav1.3$_L$ R930H channel transiently transfected in CHO cells. Representative current traces of wild-type rCav1.3$_L$ and the respective mutant rCav1.3$_L$ R930H. (**b**) Illustration of the current-voltage relationship and (**c**) the conductance-voltage relationships of wild-type rCav1.3$_L$ and mutant rCav1.3$_L$ R930H. (**d**) Analysis of the peak current densities of the wild-type rCav1.3$_L$ and mutant rCav1.3$_L$ R930H. (**e**) Representative current traces of voltage-dependence of inactivation recordings for wild-type rCav1.3$_L$ and mutant rCav1.3$_L$ R930H and (**f**) the corresponding analysis of the voltage-dependence of inactivation. Data are presented as mean ± S.E.M.

Figure S5

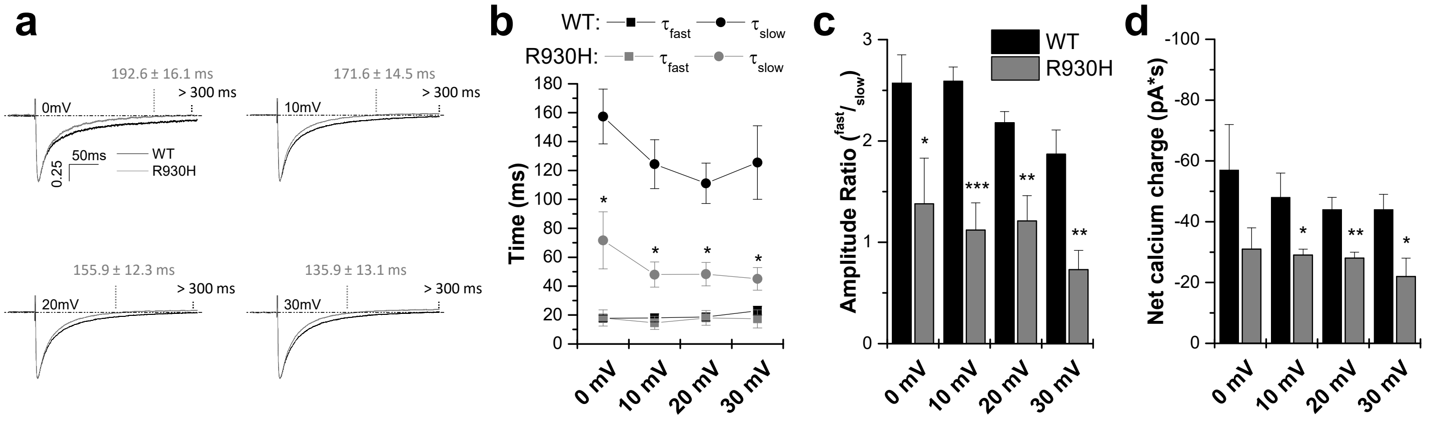

Figure S5. *R930H causes an enhanced inactivation in a long transcript variant of the rat Cav1.3 channel (rCav1.3$_L$).* (**a**) Analysis of the inactivation kinetics of wild-type rCav1.3$_L$ and mutant rCav1.3$_L$ R930H. Illustration of the time necessary to reach full inactivation for wild-type rCav1.3$_L$ and mutant rCav1.3$_L$ R930H. (**b**) Fast and slow time constants of inactivation for wild-type rCav1.3$_L$ and mutant rCav1.3$_L$ R930H, illustrating an enhanced speed of the slow time constant of inactivation for the rCav1.3$_L$ R930H. τ1 = fast component or time constant of inactivation. τ2 = slow component or time constant of inactivation. (**c**) Ratio of the amplitudes for the fast and slow components of inactivation, analyzed for wild- type rCav1.3$_L$ and mutant rCav1.3$_L$ R930H. The data indicates a reduced contribution of the fast component of inactivation. (**d**) Net Ca^{2+} flux of wild-type rCav1.3$_L$ and mutant rCav1.3$_L$ R930H, analyzed by the integral of the currents recorded at different potentials. The reduced integrals (in pA*s) indicate a significant loss-of-function caused by the mutant rCav1.3$_L$ R930H. Data are shown as mean ± S.E.M.

Figure S6

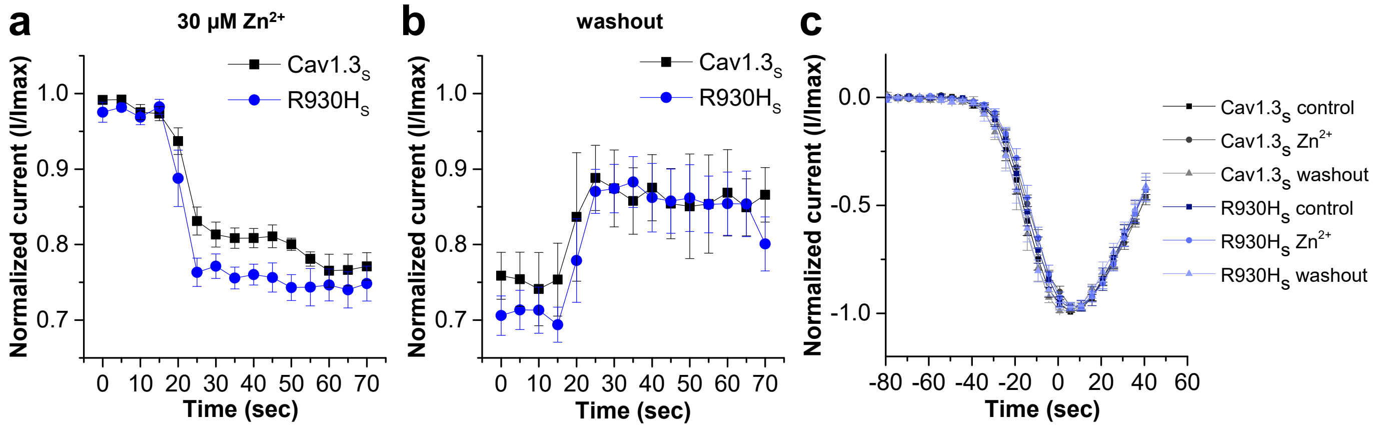

Figure S6. *Inhibition of I_{Ca} from Cav1.3$_S$ and R930H$_S$ subunits by Zn^{2+}.* (**a**) Effect of application of 30 µM Zn^{2+} on I_{Ca} mediated by Cav1.3$_S$ or R930H$_S$ upon depolarization to V_{max} at 0.2 Hz. In both mutant and wild-type channels, 30 µM Zn^{2+} significantly inhibited I_{Ca} (fraction of remaining current before Zn^{2+} application: Cav1.3$_S$: 0.99 ± 0.01, R930H$_S$: 1.01 ± 0.01 and after application Cav1.3$_S$: 0.79 ± 0.02; $p = 0.0002$, R930H$_S$: 0.77 ± 0.01, $p = 0.0009$. Statistical analysis was performed with paired Student's t-test. (**b**) I_{Ca} inhibition by Zn^{2+} was reversible upon washout with standard bath solution (see Methods). Data are shown as mean ± S.E.M., n = 5 for both constructs. (**c**) Normalized current-voltage relationships from Cav1.3$_S$ and R930H$_S$ before (n = 6 for both), upon Zn^{2+} application (n = 6 for both) and after washout (n = 5 for both). Data are shown as mean ± S.E.M..

Table S1. *Statistics of quality, mapping, coverage, depth and variant detection in each sample*

Sample ID	III_1	III_4	II_2	I_2
**Affection status**	affected	affected	affected	affected
**Total reads**	48,780,478	60,797,674	51,719,766	52,835,940
**Q30 [%]**	91.3	91.64	91.5	91.55
**Properly mapped reads [%]**	98.45	98.29	98.64	98.2
**Effective yield on target [Mb]**	4462.06	5688.75	4735.42	4666.76
**Average sequence depth on target [%]**	73.81	94.10	78.33	77.19
**Fraction of target that was covered with at least 20x [%]**	91.8	95.0	90.8	92.4
**Total variants**	306,483	359,244	323,320	352,639
SNP	265,171 (86,5%)	310,574 (86,5%)	279,503 (86,4%)	305,349 (86,6%)
InDel	41,312 (13,5%)	48,670 (13,5%)	43,817 (13,6%)	47,290 (13,4%)
**Variants total in CDS**	23,207	23,374	22918	22741
CDS SNP	22,499 (96,8%)	22,652 (96,8%)	22218 (96,9%)	22011 (96,8%)
CDS InDel	708 (3,2%)	722 (3,1%)	700 (3,1%)	731 (3,2%)
**Splice region variants total**	131	130	139	142
SNP	60 (46,2%)	59 (45,4%)	64 (46,0%)	62 (43,7%)
InDel	71 (53,8%)	71 (54,6%)	75 (54,0%)	80 (56,3%)

Table S2. *List of shared variants and genes after WES of 4 affected family members*

Gene	Disease*	Variant [c.]	Variant [p.]	MAF [%]	VarCards† [deleterious /all]	Gene Expression RPKM‡ [heart]
SSX2IP	-	116T>C (NM_014021.3)	Leu39Pro	0.01	14/23	2.4
RNF207	Long QT syndrome	995delC (NM_207396.2)	Pro332LeufsTer 44	-	-	5.9
RAB17	-	172A>G (NM_022449.3)	Lys58Glu	0.004	13/23	0.4
**CACNA1D**	**SANND, epilepsy, autism spectrum disorder, hyperaldosteronism**	**2789G>A (NM_000720.3)**	**Arg930His**	**0.05**	**21/23**	**0.2**
MYH15	-	1417G>A (NM_014981.1)	Asp473Asn	0.0057	20/22	0.2
OSBPL11	-	511C>T (NM_022776.4)	Arg171Trp	0.0049	17/23	2.8
SCAP	Myocardial infarction	1427G>A (NM_012235.2)	Arg476Gln	0.01	14/23	4.8
ABCA8	Low HDL cholesterol	3262C>T (NM_007168.2)	Arg1088Cys	0.00024	13/23	10
BPTF	Developmental delay/intellectual disability & speech delay	5237A>G (NM_182641.3)	Asn1746Ser	0.00004	12/22	3
HDHD2	-	677G>A (NM_032124.4)	Gly226Glu	-	18/22	5
MED16	-	-18-1G>A (NM_005481.2)	?	-	-	2.8
TCF3	Hypogammaglobulinaemia and acute lymphoblastic leukaemia	1313G>A (NM_003200.3)	Arg438Gln	0.0049	14/23	1.6
IDH3G	-	167C>T(NM_004135.3)	Thr56Met	0.0016	14/21	18.4
MECP2	Rett syndrome	851C>T (NM_001110792.1)	MECP2	0.0007	19/21	4.2

*associated disease in HGMD or ClinGen; †ratio of damaging/deleterious prediction in *in silico* algorithm; ‡ according to HPA RNA-seq normal tissue database; SANDD, sinoatrial node dysfunction and deafness; RPKM, reads per kilo base per million mapped reads
